# Meta-analysis and systematic review of vesicular monoamine transporter (VMAT-2) inhibitors in schizophrenia and psychosis

**DOI:** 10.1007/s00213-023-06488-3

**Published:** 2024-01-19

**Authors:** Anne Connolly, Phoebe Wallman, Olubanke Dzahini, Oliver Howes, David Taylor

**Affiliations:** 1 Pharmacy Department, Maudsley Hospital, London, SE5 8AZ UK; 2 Institute of Psychiatry, Psychology and Neuroscience, King’s College London, De Crespigny Park, London, SE5 8AF UK; 3 H Lundbeck A/s, 3 Abbey View, Everard Close, St Albans, AL1 2PS UK; 4 Institute of Clinical Sciences (ICS), Faculty of Medicine, Imperial College London, Du Cane Road, London, W12 0NN UK; 5 Institute of Pharmaceutical Science, King’s College London, Stamford Street, London, SE1 9NH UK

**Keywords:** Psychosis, Dopamine, VMAT inhibitor, Tardive dyskinesia, Supersensitivity

## Abstract

**Rationale:**

Dopamine antagonists induce dopamine receptor supersensitivity. This may manifest in late-appearing movement disorders (tardive dyskinesia (TD). VMAT-2 inhibitors reduce dopaminergic transmission but have limited activity at postsynaptic receptors and so may have antipsychotic activity with lower risk of tardive dyskinesia.

**Methods:**

We conducted a systematic database search from inception to September 2022 for articles describing the use of VMAT-2 inhibitors in psychosis. Inclusion criteria were as follows: *Population*: adults diagnosed with psychosis or schizophrenia; *Intervention*: treatment with tetrabenazine, deutetrabenazine or valbenazine; *Comparison*: comparison with placebo or/and antipsychotic drug; *Outcomes*: with efficacy outcomes (e.g. Brief Psychiatric Rating Scale (BPRS) change or clinician assessment) and adverse effects ratings (e.g. rating scale or clinician assessment or dropouts); and *Studies*: in randomised controlled trials and non-randomised studies.

**Results:**

We identified 4892 records relating to VMAT-2 inhibitor use of which 5 (173 participants) met our a priori meta-analysis inclusion criteria. VMAT-2 inhibitors were more effective than placebo for the outcome ‘slight improvement’ (risk ratio (RR) = 1.77 (95% CI 1.03, 3.04)) but not for ‘moderate improvement’ (RR 2.81 (95% CI 0.27, 29.17). VMAT-2 inhibitors were as effective as active comparators on both measures for—‘slight improvement’ (RR 1.05 (95% CI 0.6, 1.81)) and ‘moderate improvement’ (RR 1.11 (95% CI 0.51, 2.42). Antipsychotic efficacy was also suggested by a narrative review of 37 studies excluded from the meta-analysis.

**Conclusions:**

VMAT-2 inhibitors may have antipsychotic activity and may offer promise for treatment of psychosis with the potential for a reduced risk of TD.

**Supplementary Information:**

The online version contains supplementary material available at 10.1007/s00213-023-06488-3.

## Introduction

It is accepted that the symptoms of schizophrenia are at least in part caused by abnormalities in neuronal dopamine function. The core dysfunction appears to be an increase in presynaptic synthesis and release of striatal dopamine (DA) (Howes et al. 2017). Most currently available antipsychotics block postsynaptic dopamine 2 (D2) receptors (pimavanserin is the sole exception (Rissardo et al. 2022)) but do not significantly alter dopamine synthesis capacity at contemporary therapeutic doses (Jauhar et al. 2019). D2 antagonism may induce sensitivity of postsynaptic dopamine receptors (Chouinard et al. 1978; Fallon et al. 2012), and cessation of D2 antagonists may then expose the patient to the effects of both the inherent elevated DA synthesis and supersensitive postsynaptic D2 receptors. This drug-induced sensitivity and the absence of effect on the core pathological mechanism help explain the very high risks of relapse after stopping antipsychotics (90% at 2 years after a first episode) (Zipursky et al. 2014).

There are several potential pharmacological methods of countering increased synthesis of dopamine. These include directly decreasing synthesis, reducing intracellular transport, inhibiting vesicular storage, stimulating pre-synaptic (negative feedback) receptors and blocking post-synaptic receptors. Of these methods, only post-synaptic D2 antagonism is used clinically (although pre-synaptic blockade may also contribute via depolarization block) (Grace et al. 1997). However, there are licensed compounds available which affect vesicular monoamine uptake. Deutetrabenazine, tetrabenazine and valbenazine deplete vesicular storage of dopamine (as well as serotonin, noradrenaline and histamine) in presynaptic nerve terminals by reversibly inhibiting human vesicular monoamine transporter isoform 2 (VMAT-2 inhibitors). This inhibition results in a reduced inclusion of monoamines into synaptic vesicles, a corresponding depletion of functional monoamine stores and ultimately a reduction in neurotransmitter release. The three available VMAT-2 inhibitors have a broadly similar mode of action. Most is known about tetrabenazine—a drug that is more than 60 years old. Tetrabenazine preferentially depletes dopamine over serotonin and noradrenaline whilst also modestly reducing levels of acetylcholine, glutamate and aspartate (Stahl 2018b; Zheng et al. 2006).

VMAT-2 inhibitors do not appear to induce postsynaptic DA supersensitivity. Indeed, VMAT-2 inhibitors are used to treat tardive dyskinesia (TD)—a condition caused by chronic dopamine receptor blockade and a presumed consequent upregulation of D2 receptors and altered synaptic plasticity. Moreover, by reducing presynaptic dopamine storage and thus the amount released into the synaptic cleft, VMAT-2 inhibitors act more directly against excess stimulation of hypersensitive D1 and D2 receptors, the proposed pathological basis of TD.

VMAT-2 inhibitors are licensed only for the treatment of TD but tetrabenazine was at one time indicated as an antipsychotic. These drugs may offer the prospect of improved outcome in long-term treatment of psychosis; they may reduce the severity of psychotic symptoms but with a more limited effect on postsynaptic DA receptor sensitivity. We undertook a systematic review of studies of VMAT-2 inhibitors in people with psychosis and similar conditions.

## Method

### Meta-analysis

#### Study selection

##### A priori selection parameters

The criteria for inclusion in this review were as described in the following pre-defined PICOS tool. *Population*: adults (minimum of 18 years old, including older adults aged more than 65 years old) who were diagnosed with psychosis or schizophrenia (or broad equivalent in older studies). The diagnostic method was not restricted in any way. *Intervention*: treatment with dopamine synthesis inhibitors: tetrabenazine, deutetrabenazine or valbenazine. Any route of administration, dose, duration and co-intervention treatment was eligible. *Comparison*: placebo or/and antipsychotic drug. *Outcomes*: efficacy (e.g. Brief Psychiatric Rating Scale (BPRS) change or clinician assessment) and adverse effects (e.g. rating scale or clinician assessment or dropouts). *Studies*: randomised controlled trials and non-randomised studies.

Studies that did not meet the criteria listed above were examined for inclusion in a narrative review.

##### Search methods for identification of studies

The following databases were searched: EMBASE, EMBASE Classic, MEDLINE, PsycINFO, Psychiatry Online, PubMed and Web of Science. We also included all the citations from the top 10% of references in the Web of Science search.

All the databases (except PubMed) were searched from inception until November 2020 using the keyword terms below. Search terms were adapted to the thesaurus of each database.

**Table:** 

Boolean operator	Search term
OR	schizo* “dementia praecox” psychos?s psychotic
AND	
OR	valbenazine deutetrabenazine tetrabenazine “dopamine synthesis inhibitor*” “vesicular monoamine transport inhibitor*”

These databases including PubMed were also searched for a second time using the terms ‘Nitoman’ and ‘R1-9569’ from inception until May 2021. This entire search was repeated for the last time in September 2022.

Medical Subject Headings (MeSH) terms were used where available; otherwise, free-text searching was used. Studies were limited to humans, and there were no restrictions in respect to language. Google Translate and native speakers were used to translate non-English language studies.

The reference sections of all studies retrieved were also searched for relevant studies.

#### Data collection and analysis

##### Selection of studies

Three authors (DT, AC and PW) screened study abstracts and titles and retrieved potentially relevant complete text papers for detailed examination. Disagreements about inclusion were resolved by discussion with a fourth author (OD).

##### Data collection and management

Two authors (AC and PW) extracted study data independently, and disagreements between the two were resolved by discussion with a third author (OD). For studies where relevant data were not available, the study authors were contacted via email. The following study characteristics were extracted for the meta-analysis section of this investigation: year of publication, country of origin, study design, main diagnosis, duration of treatment, number of participants (total, responders, remitters, dropouts for intervention and comparator), age range of participants, care setting, sex, intervention, comparator and outcome measure (baseline scores (SD) and endpoint score (SD) for intervention and comparator). Study-defined psychosis rating scale scores were extracted to determine response to treatment. Response was categorised as ‘slight improvement’ and ‘moderate improvement’, corresponding to Clinical Global Impressions Improvement Scale (CGI-I) scores 3 (minimally improved) and 2 (much improved), respectively. For example, ‘definite improvement’, ‘significant improvement’ or ‘marked improvement’ were categorised as CGI-I 2 and ‘slight improvement/response’ as CGI-I 3 (See supplementary Table S2).

Study authors were contacted to obtain missing information or to clarify the information available (AC).

##### Assessment of risk of bias in included studies

The Cochrane risk of bias tool (version 2) (Sterne et al. 2019) was completed independently by two authors (AC and PW), with disagreements resolved by a third author (OD). The assessment of the risk-of-bias was incorporated into the interpretation of the results.

##### Dealing with missing data and assessment of reporting biases

The impact of missing data in individual studies was explored by imputing missing values with reported reasons for their ‘missingness’ and synthesising these using STATA’s METAMISS command (White and Higgins 2007).

##### Assessment of heterogeneity

The presence and extent of heterogeneity were explored by visual inspection of forest plots and quantified using the *I*^2^ statistic. Funnel plots were constructed where possible to assess for missing studies.

##### Data synthesis, measures of treatment effect and unit of analysis issues

We performed a random-effects model using Der Simonian and Laird weights to synthesise results. We used risk ratios as the effect measure of interest with corresponding 95% confidence intervals and displayed results using forest plots. Separate analyses were conducted for those achieving at least slight or moderate improvement in psychotic symptoms. Crossover studies were assessed for evidence of carryover effects. If carryover effects were not present, then both periods were covered; otherwise, studies were analysed as parallel studies by using results only from the first period. When both periods from a crossover study were included, Becker–Balagtas estimates were computed and combined with odds ratios from parallel studies (Stedman et al. 2011). Meta-analysis and forest plots were conducted on Revman V 5.4 (Collaboration. 2020).

##### Sensitivity analyses

We performed different sensitivity analyses to assess robustness of the primary methods by varying analysis by effect size, model type, crossover study periods and missing observation imputation.

##### Narrative review

*Post hoc*, we decided to provide a narrative review of informative reports which did not meet inclusion criteria for the meta-analysis. We included in this review any report of the use of any VMAT-2 inhibitor in at least one person with a diagnosis of schizophrenia or psychosis (or any accepted symptom of psychosis) and where any evaluation of psychosis symptom severity was made.

## Results

### Meta-analysis

**Fig. 1:**
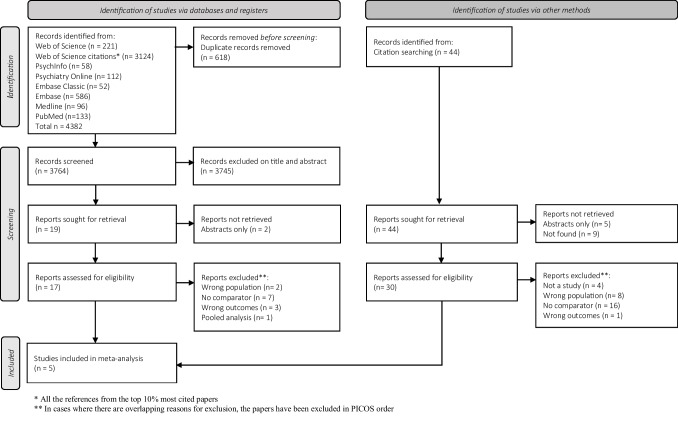
Study selection (flow of studies)

The results of the search and selection process of studies are described in the PRISMA flow diagram (Fig. 1). The search identified 4892 records of which five met the inclusion criteria of our pre-defined PICOS. The characteristics of included studies are shown in Table 1. Included studies were from Europe and North America and spanned a 52-year period. Only tetrabenazine studies were included in the meta-analysis as these were the only VMAT-2 inhibitor studies that fitted our inclusion criteria.

**Table 1:** Meta-analysis study characteristics

*Author*	*Year*	*Country*	*Design*	*Main diagnosis*	*Number of participants (combined, before dropouts)*	*Mean age (SD)*	*Care setting*	*Males (%)*
Ashcroft, G. W., MacDougall E. J. and Barker, P. A. (1961)	1961	Scotland	Non-randomised trial, double-blind	Schizophrenia	52	Tetrabenazine 56.1 (12.8); chlorpromazine 58.3 (11.0)	Inpatients	0 (0)
Lingjaerde, O. (1963)	1963	Norway	Randomised controlled trial cross over	Schizophrenia and psychosis, autism (and were mute), had at least 1 previous antipsychotic failure (chronically ill, several previous antipsychotics)	25 (24 with schizophrenia)	32–85 mean not stated or SD	Inpatients	0 (0)
Remington, G., Kapur, S., Foussias, G., Agid, O., Mann, S., Borlido, C., Richards, S. and Javaid, N. (2012)	2012	Canada	Randomised controlled double-blind trial, intention to treat population	Treatment resistant psychosis, patients maintained on current antipsychotic mostly clozapine	41	43 (10)	Outpatient	32 (78)
Smith, M. E. (1960)	1960	USA	Non-randomised, double-blind trial	Schizophrenia	35	38 M, 40 F	Inpatient	10 (29)
Weckowicz, T.E., Ward, T. and Hoffer, A. (1960)	1960	Canada	Non-randomised double-blind trial, pilot study	Schizophrenia	20	Not stated	Inpatient	20 (100)
*Author*	*Year*	*Intervention*		*Comparator*	*Tetrabenazine dose (mg/day)*		*Duration (weeks)*	*Outcome measure*
Ashcroft, G. W., MacDougall E. J. and Barker, P. A. (1961)	1961	Tetrabenazine		Chlorpromazine (CPZ)	90mg for 4 weeks, 120mg for 8 weeks		20 (6 weeks washout then 2 weeks placebo and 12 weeks active treatment)	Baker and Thorpe*, clinical assessment
Lingjaerde, O. (1963)	1963	Tetrabenazine		Placebo (some drop out cases were on 2 antipsychotics concurrently)	150mg		6 (3 placebo and 3 weeks active treatment)	Clinician assessment
Remington, G., Kapur, S., Foussias, G., Agid, O., Mann, S., Borlido, C., Richards, S. and Javaid, N. (2012)	2012	Tetrabenazine augmentation of clozapine (and other antipsychotics)		Placebo augmentation of clozapine	12.5–75mg		12	Brief Psychiatric Rating Scale (BPRS), Clinical Global Impression (CGI), Global Assessment of Functioning (GAF), Behaviourally Anchored Rating Scale (BARS), Riker Sedation-Agitation Scale (SAS), Calgary Depression Scale for Schizophrenia (CDS)
Smith, M. E. (1960)	1960	Tetrabenazine		Reserpine or placebo	30–300mg		10 to 16	Clinician assessment, Behavioural Rating Scale
Weckowicz, T.E., Ward, T. and Hoffer, A. (1960)	1960	Tetrabenazine		Placebo	100mg		2	Weyburn Assessment Scale**, clinician assessment

*Baker and Thorpe Behaviour Rating Scale includes ten behavioural items. It is used to measure (a) schizophrenic deterioration by adding the daily totals for the ten items on the scale to give a weekly score and (b) restlessness, the sum of weekly totals for day and night restlessness (2 items) as a measure of psychomotor restlessness

**Weyburn Assessment Scale evaluated behavioural changes in chronic hospitalised patients with schizophrenia by using seven behavioural categories (appearance, behaviour, thought, perception, mood, memory and insight) to assess their current treatment

There was a total of 173 participants (before dropouts) allocated to tetrabenazine, active comparator or placebo in the included studies. Four of the studies (108 participants) used placebo as a comparator and two (76 participants) used active comparators (chlorpromazine or reserpine). One of these studies (Remington et al. 2012) used tetrabenazine or placebo augmentation of clozapine (or other antipsychotics) whilst another (Smith 1960) used both an active (reserpine) and a placebo comparator. Study responder categorisation by CGI-I score is described in the method and in detail in Supplementary Table S2. No data were available on numbers of patients achieving remission. Dropouts were reported for two of the placebo comparison studies and one of the active studies; the total number of dropouts was 23.

### Study characteristics of included studies

See Table 1.

### Synthesis

**Fig. 2:**
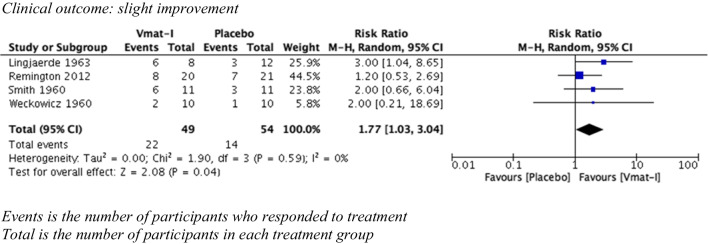
Clinical outcome vs placebo: slight improvement

**Fig. 3:**
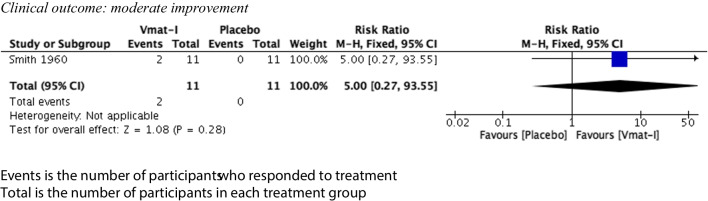
Clinical outcome vs placebo: moderate improvement

**Fig. 4:**
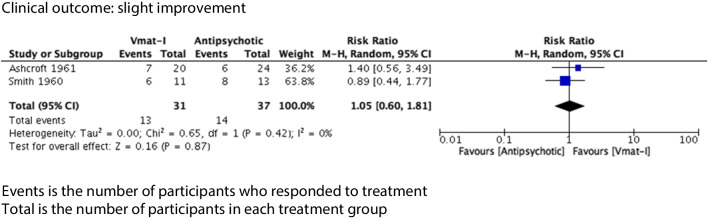
Clinical outcome vs active comparator: slight improvement

**Fig. 5:**
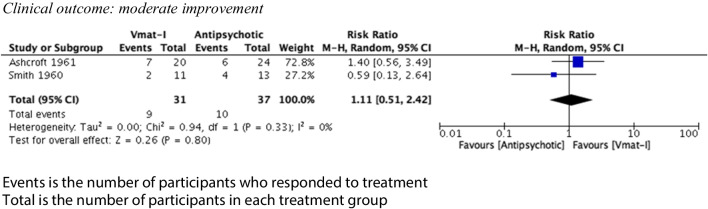
Clinical outcome vs active comparator: moderate improvement

Clinical outcomes were divided into two categories, slight improvement and moderate improvement, as described. For VMAT-2 inhibitors versus placebo (see Figs. 2 and 3), there was a statistically significant advantage for the clinical outcome slight improvement (4 studies, 103 participants) (risk ratio (RR) 1.77 (95% CI 1.03, 3.04) but no advantage for the clinical outcome moderate improvement (RR) 5.00 (95% CI 0.27, 95.33). Both syntheses had no statistical heterogeneity (*I*^2^ = 0%). Sixty-eight participants (31 VMAT-2 inhibitors, 37 active comparators) were included in analysis comparing VMAT-2 inhibitors with an active comparator (see Figs. 4 and 5). For VMAT-2 inhibitors versus active comparator, see Figs. 4 and 5. There was no statistically significant difference for the clinical outcome ‘slight improvement’ (RR 1.05 (95% CI 0.6, 1.81)) or ‘moderate improvement’ (RR 1.11 (95% CI 0.51, 2.42). Both syntheses had no statistical heterogeneity (*I*^2^ = 0%).

Sensitivity analyses were performed by repeating the analyses with imputed missing outcome data, OR as effect size measure and inclusion of both periods in the crossover study (Figures S1, S2). Results of all sensitivity analyses were not significantly different to the original analyses except for inclusion of both periods of the crossover study which resulted in slightly larger effect (OR 3.45; 95% CI 1.16, 8.47) compared with analysis from the first period only (OR 2.45 95% CI 1.03, 5.87). The direction of effects did not change in any of the analyses (see figure S2).

We could not assess for publication bias visually using funnel plots because of the small number of studies included.

### VMAT-2 inhibitors versus placebo

See Fig. 2

### VMAT-2 inhibitors versus active comparator

See Fig. 4

### Risk of bias

The Cochrane risk-of-bias assessments are summarised in Tables 2 and 3. One study was assessed as being at a low risk of bias; three had some concerns, and one had a high risk of bias (elaboration of these concerns are detailed in Table S3). No studies were excluded by risk-of-bias.

**Table 2:** Summary of Cochrane risk-of-bias tool for randomised trials (version 2) per protocol

Study	Randomisation process	Deviations from intended interventions	Missing outcome data	Measurement of outcome	Selection of reported result	Overall bias
Ashcroft et al. (1961)	Some	Low	Low	Low	Some	Some
Lingjaerde (1963)	Low	High	Low	Low	Some	High
Smith (1960)	Some	High	Low	Some	Some	High
Weckowicz et al. (1960)	Some	Low	Low	Some	Some	Some

**Table 3:** Summary of Cochrane risk-of-bias tool for randomised trials (version 2) intention to treat

Study	Randomisation process	Deviations from intended interventions	Missing outcome data	Measurement of outcome	Selection of reported result	Overall bias
Remington et al. (2012)	Low	Low	Low	Low	Low	Low

### Synthesis studies—adverse events

Adverse effects reported in studies included in the systematic review are described in Tables 4 and 5.

**Table 4:** Adverse reactions in systematic review: placebo studies. NI, no information

Study	Adverse reaction descriptor	Intervention tetrabenazine			Comparator (placebo)			Comments
		*N* total frequency	*N* dropout due to adverse reaction total	*N* total patients with adverse reactions	*N* total frequency	*N* dropout due to adverse reaction total	*N* total patients with adverse reactions	
Lingjærde (1963)	Drowsiness	14	5	16	NI	0	1	Numbers uncertain owing to unclear description in original report
	Extrapyramidal side effects including dystonia	3			NI			
	Anginal pain and extrasystoles	1			NI			
	Urine incontinence	1			NI			
	Increased appetite	1			NI			
	Abdominal pain	1			NI			
	Death (bronchopneumonia; cerebral thrombosis)	2			NI			
Remington et al. (2012)	Parkinsonism	NI	0	NI	NI	NI	NI	Dropouts due to adverse reactions in placebo group not stated in text; no significant difference between tetrabenazine and placebo (judged from rating scale assessments)
	Depression	NI			NI			
	Sleepiness/sedation	NI			NI			
	Reduced duration of sleep	NI			NI			
	Tension/inner unrest	NI			NI			
	Asthenia/lassitude/fatiguability	NI			NI			
	Orthostatic dizziness	NI			NI			
	Systolic BP	NI			NI			
	Weight gain	NI			NI			
Smith (1960)	Drowsiness	5	NI	8	NI	NI	0	Dropout numbers due to adverse reactions not listed for placebo or tetrabenazine
	Parkinson-like-syndrome	3			NI			
	Drooling	4			NI			
	Loss of equilibrium	1			NI			
Weckowicz et al. (1960)	Overactivity problem	2	NI	NI	NI	NI	NI	Dropout numbers not listed No change in blood pressure, weight or white blood cell count observed
	Hot flushes	2			NI			
	Drowsiness	3			NI

**Table 5:** Adverse reactions in systematic review: active comparator studies. NI, no information

Study	Adverse reaction descriptor	Intervention tetrabenzine			Comparator			Comments
		*N* total frequency	*N* dropout due to adverse reaction total	*N* total patients with adverse reactions	*N* total frequency	*N* dropout due to adverse reaction total	*N* total patients with adverse reactions	
Ashcroft et al. (1961)	Subacute delirium	2	5	13?	NI	0	9?	One chlorpromazine comparator patient had cerebral thrombosis during placebo treatment; tetrabenazine—significant diastolic blood pressure fall; chlorpromazine—significant diastolic and systolic blood pressure fall and pulse rate increase
	Generalised tremor	1			1 (fine tremor)			
	Marked unreality feelings and agitation	1			NI			
	Suicidally depressed	1			NI			
	Catatonic stupor	1			NI			
	Parkinsonism syndrome	1			4			
	Extremely drowsy	2			4 (drowsiness)			
	Parkinson-like-syndrome	4			NI			
Smith (1960)	Drowsiness	5	NI	8	3	NI	6	Dropouts due to adverse reactions not listed for tetrabenazine or reserpine
	Parkinson-like-syndrome	3			3			
	Drooling	4			0			
	Loss of equilibrium	1			2			

### Narrative review studies excluded from the meta-analysis

We identified 37 studies which did not meet our pre-defined criteria for inclusion in the meta-analysis but were considered informative. Our inclusion criteria were as described in the ‘Methods’ section. These studies were published over a period of 64 years (1958–2022). Tardive dyskinesia was co-existent with schizophrenia in seven of the 37 studies (Hauser et al. 2017; Josiassen et al. 2017; Kalian et al. 1993; Khurram et al. 2021; Lindenmayer et al. 2022; Lindenmayer et al. 2017; Lindenmayer et al. 2019); seven studies were placebo-controlled (Ashcroft et al. 1961; Hauser et al. 2017; Lindenmayer et al. 2017; Lingjaerde 1963; Remington et al. 2012; Smith 1960; Weckowicz et al. 1960). One study substituted reserpine (Brauchitsch) as a means of comparison. Treatment duration for tetrabenazine ranged from 2 days to 22 months and daily doses from 10 to 600mg (most were in the range 100–200mg/day) and for valbenazine from 3 to 48 weeks, at 25 to 80mg/day.

For many studies involving tetrabenazine (Table 6), it was somewhat unclear what prior or concurrent treatment had been or was given. Treatments reported as being prescribed before or alongside tetrabenazine included antipsychotics, reserpine, ECT, iproniazid, imipramine, barbiturates, promethazine, insulin, lobotomy and ‘special drug therapy’. ECT was employed as concurrent co-therapy in at least three studies (Lustig 1961; Shimizu et al. 1962; Voelkel and Dressler 1959). Two reports explicitly excluded the co-prescription of all other treatments (Borenstein et al. 1961; Heinze 1960).

**Table 6:** Studies included in the narrative review—tetrabenazine

Author(s)/year	Tetrabenazine dose (route)	Number of patients	Duration of treatment	Condition	Study outcome for SCZ/psychosis cases
Voelkel (1958)	90–450mg only (optimal dose 90–120mg PO), parenteral dose unclear—up to 240mg	Tetrabenazine alone, *n* = 64, tetrabenazine + prior iproniazid, *n* = 58	Not stated	Schizophrenia (*n* = 33) and delusional psychosis in menopause (*n* = 31); paranoid hallucinatory conditions (*n* = 22), delirium in senile psychosis (*n* = 16), agitated anxious depressives (*n* = 14), anxiety, OCD disorders (*n* = 6)	Unclear, implied positive effect by authors of study
Haug and Stenstad (1959)	60–105mg (not stated)	26	2–12 w	Schizophrenia	11/26 some improvement
Lingjaerde (1959)	30–100mg (not stated)	25	56 d	Schizophrenia	Some benefit in 12/25
Voelkel and Dresler (1959)	90–450mg (PO or IM or both)	29	8 d–21 m	Cerebral atrophic process with delusions (*n* = 18), psychosis (*n* = 4), other (*n* = 7)	5/24 remitted, 10/24 responded
Flegel (1960)	10–150mg (PO, 1 case IM)	89	2–40 w	Schizophrenia (*n* = 71), hebephrenia (*n* = 7), other (*n* = 11)	45/78 improved significantly, 11 effect not reported
Heinze (1960)	350mg/day (IM), then 180mg/day (PO)	28	8w–8 m	Schizophrenia (*n* = 13), hebephrenic and dementia simplex (*n* = 11), other (*n* = 4)	15/28 good or modest effect
Lende (1960)	Average 100–200mg (50–300mg) (not stated)	65	Not stated	learning disability +/−schizophrenia	52/65 some improvement
Montagut (1960)	25–200mg (not stated)	40	1 m	Schizophrenia	15/40 remission
Narros and Carranza (1960)	average 100mg (75–150mg) (not stated)	40	45 d	Schizophrenia (*n* = 37), other (*n* = 3)	37/40 remission
Pomme et al. (1960)	Average 120–180mg (90–360mg) (PO)	35	5d–2 m	Hallucinatory syndromes with delusions (*n* = 15), schizophrenia (*n* = 4), senile psychosis (*n* = 3), other (16)	16/22 good or very good outcome
Reda and Germano (1960)	Average (150mg) (start dose 100mg IM or 60mg PO to 330mg (IM+PO))	28	Average 60 d, 15 d–11 m	Schizophrenia (*n* = 23), psychoneuroses (*n* = 5)	21/28 positive response
Sacerdoti (1960)	50–150mg PO, 40–300mg IM	47	10–80 d	Schizophrenia (*n* = 26), epilepsy (*n* = 12), other (9)	27/32 some response
Schmitt (1960)	60–180mg PO	28	5 w	Psychosis	11/28 remission
Stockhausen (1960a)	Max 600mg (not stated)	48	3–4 w	Schizophrenia	Unclear, no figures provided in study
Stockhausen (1960b)	Average 75–600mg (max M 600mg, F 180mg) (IM, IV, SC, PO)	70	15 d–2 m	Schizophrenia/psychosis (*n* = 67), other (*n* = 3)	56/67 greatly improved
Stockhausen (1960c)	Average F 75mg, max 150mg; average M 75mg, max 600mg (IM, IV, PO)	114	Average of 3–5 w	Psychosis (*n* = 107), other (*n* = 7)	83/107 significantly improved
Stumpf (1960)	150–300mg (PO, occasional injection)	36	2–51 d (average 14 d)	Depression (*n* = 26 (*n* = 11 bipolar)), schizophrenia (*n* = 4), other (*n* = 6)	10/36 good remission
Bertolotti (1961)	55mg (25mg IM + 30mg PO)–300mg (PO); average 120–150mg M; 100–120mg F	29	‘Around’ 20–30 d	Schizophrenia (*n* = 20), other (*n* = 9)	15/20 much improved
Borenstein et al. (1961)	50–150mg (PO/IM)	105	15 d to not stated	Schizophrenia (*n*=25), other, i.e. mania, chronic delirium, reactive depression, psychoneurosis, ‘delusional flash’, melancholy (*n* = 80)	5/25 improved, 11/25 worse
Espinosa (1960)	50–150mg (PO/IM), 150mg optimal dose	39	22.8 d (mean), range 10 d–3 m	Schizophrenia (*n* = 18), other (*n* = 21)	12/18 total/partial remission
Lustig (1961)	75–300mg (not stated)	131	4–8 m (range 4 w–22 m)	Schizophrenia (*n* = 95), other (*n* = 36)	82/95 good or moderate improvement
Singer et al. (1961)	60–150mg (not stated)	13	3–7 w	Schizophrenia (*n* = 7), psychosis (*n* = 6)	Hallucinolytic effect, no numbers given
Kammerer et al. (1962)	90–135mg average, max 150mg (not stated)	29	3–7 w	Schizophrenia (*n* = 17), delusional flashes (*n* = 8), psychosis (*n* = 4)	Hallucinolytic effect, no numbers given
Shimizu et al. (1962)	75–250mg; mean 190.4mg (SD 40.2) (not stated)	13	23–71 d, mean 56.5 d (SD 14.1)	Schizophrenia	8/13 good or extremely good response
Burckard et al. (1962)	50–150mg (PO/IM)	36	Not stated	Schizophrenia and psychosis (*n* = 35), other (*n* = 1)	14/36 symptoms regressed/improved
Cwynar et al. (1962)	150–400mg (PO/injection)	17	16–69 d	Schizophrenia (*n* = 15), other (*n* = 2)	11/16 significant improvement or improvement
Brauchitsch (n.d., pre-1963)***	150–225mg (PO/IM)	134	8–30 d	Schizophrenia and psychosis	58/104 some, moderate or marked improvement
Lingjaerde (1963)	40–100mg, 60mg optimal dose (not stated)	34	8 w	Schizophrenia	12/25 improved moderately
Matsumoto et al. (1966)	25–150mg (PO/injection)	82	4–96 d	Schizophrenia (*n* = 67), other (*n* = 15)	28/67 effective
Kalian et al. (1993)	200mg (PO)	1	2 m	Schizophrenia with tardive dyskinesia	1/1 psychotic symptoms improved
Gordon et al. (1998)	75mg (not stated)	1	16 m	Schizophrenia	1/1 violence but not psychosis improved

***Placebo substitution in 23 pts

PO, oral; IM, intramuscular; IV, intravenous; SC, subcutaneously; M, male; F, female; d, days; w, weeks; m, months

Observations from these studies of tetrabenazine, most of which did not employ a contemporary comparator, suggested some antipsychotic action—most patients improved to some extent. In patients with schizophrenia or psychosis, tetrabenazine was moderately or greatly effective in, broadly speaking, three quarters of those treated. In one report, tetrabenazine was ineffective (Borenstein et al. 1961), and there was uncertain efficacy in a further four studies (Kammerer et al. 1962; Singer 1961; Stockhausen 1960c; Voelkel 1958).

Symptoms reported to have responded well included aggression and violence, negativity, restlessness, paranoia, delusions and hallucinosis. Conversely, autism, apathy, insomnia and lack of insight appear to have responded relatively less well. In the study in which some of the tetrabenazine-treated patients were switched to placebo (Brauchitsch), 14 of 23 relapsed after switching.

The most commonly reported adverse effects of tetrabenazine were parkinsonism (tremor, dystonia, akathisia/restlessness) and hypotension, both of which were noted to be dose-related. Depression was specifically mentioned as being observed in two studies and dysphoria (as part of ‘tetrabenazine malaise’ syndrome) in one. Suicidal ideation was not mentioned in any report. Gastrointestinal effects such as nausea (with resulting anorexia), vomiting, diarrhoea and constipation were commonly reported. Somnolence was also usually seen. Other reported adverse effects included insomnia and sleep disturbance. One study described the (apparently then well-known phenomenon) of ‘tetrabenazine malaise’—a syndrome consisting of dysphoria, irritability, fatigue and lethargy. More than one study reported the occurrence of ‘neuroleptic syndrome’ but without further clarification or description. Weight gain was reported in some studies. Many trials reported the occurrence of either dry mouth or hypersalivation, some tachycardia and some bradycardia.

The extent of participant withdrawal from treatment because of adverse effects was not described in most studies. Completion rates were generally very high when reported, but, given the age of most of the studies, it is likely possible that participants had little choice but to continue even when there was poor tolerability. Indeed, whilst most patients received oral medication, some studies administered tetrabenazine covertly or parenterally in those patients who refused treatment.

The six most recent studies examined the use of valbenazine in participants with both tardive dyskinesia and schizophrenia, but in each of these, the intention was to test efficacy in reducing the severity of TD (Table 7). The subjects included were chronically, but not acutely unwell and schizophrenia symptom severity change was not anticipated. Valbenazine was added to on-going antipsychotic treatment in five of these studies (Hauser et al. 2017; Josiassen et al. 2017; Khurram et al. 2021; Lindenmayer et al. 2017; Lindenmayer et al. 2019). Symptom ratings suggested that participants’ psychiatric illness remained stable during valbenazine treatment with negligible, albeit favourable, changes in PANSS scores that were not statistically different from those with placebo. There was no evidence from these trials that valbenazine had any worthwhile antipsychotic activity when added to existing antipsychotic treatment. However, this was not the objective of these studies; rather, they were safety studies designed in part to assess any potential detrimental adverse effects on participants’ mental state. Two recent case studies described apparent antipsychotic effects of valbenazine in treatment-resistant schizophrenia with TD: one for a patient not taking an antipsychotic (Lindenmayer et al. 2022) and the other taking clozapine (Khurram et al. 2021).

**Table 7:** Studies included in the narrative review—valbenazine

*Author(s)/year:*	*Valbenazine dose (route):*	*Number of patients:*	*Duration of treatment:*	*Condition:*	*Study outcome for SCZ/psychosis cases*
Hauser et al. (2017)*	40mg or 80mg (PO)	150	6 w	Schizophrenia, schizoaffective disorder, mood disorder with tardive dyskinesia	No deterioration in mental state for 86 cases vs. PBO 43 (completers)
Josiassen et al. (2017)	40mg or 80mg (PO)	309	48w	Schizophrenia, schizoaffective disorder, mood disorder al with tardive dyskinesia	Psychiatric status remained stable
Lindenmayer (2017)*	25–75mg (PO)	100	6 w	Schizophrenia, schizoaffective disorder, mood disorder al with tardive dyskinesia	Psychiatric status remained stable
Lindenmayer (2019)	40–80mg (PO)	163	48 w	Schizophrenia, schizoaffective disorder, mood disorder al with tardive dyskinesia	Psychiatric status remained stable
Khurram et al. (2021)	80mg	1	6 w+	Schizophrenia, tardive dyskinesia	Improvement of negative symptoms
Lindenmayer et al. (2022)	80mg	1	3 w+	Schizophrenia, Lewy body dementia depression with psychosis, tardive dyskinesia	Improvement of positive symptoms

*Randomised-controlled trial

PBO, placebo; d, days; w, weeks; m, months

Valbenazine adverse effects in studies included in this review included somnolence, akathisia, dry mouth, urinary tract infection and headache, also arthralgia, headache, vomiting, anxiety, insomnia, fatigue, urinary tract infection and weight gain. Data on suicidal ideation differed—one study recorded lower rates than with placebo whilst another found the converse effect (possibly explained in this latter study by a higher baseline level in the valbenazine group).

Studies of the effect of deutetrabenazine in TD (Anderson et al. 2017; Fernandez et al. 2017) examined mixed populations of individuals, only some of whom had psychotic illness. The effect of deutetrabenazine on psychotic symptoms was not formally evaluated. Adverse effect incidence did not differ from placebo.

## Discussion

This meta-analysis tentatively suggests tetrabenazine has some antipsychotic efficacy, although its relative efficacy compared with dopamine antagonists could not be determined. Our narrative review (albeit of studies of dubious quality) also suggests that VMAT-2 inhibitors are effective as antipsychotics. The major obstacle in clarifying the exact utility of VMAT-2 inhibitors in psychosis is the near absence of trials which meet today’s methodological standards. The number of studies meeting our inclusion criteria was small, and the power to reveal differences between treatments was correspondingly low. In the narrative review, study quality was poor with limited or confused description of trial design, little or no description of blinding, no or limited statistical analysis and widespread use of unvalidated rating scales and other assessments. Although valbenazine and deutetrabenazine have been evaluated in modern, well-conducted studies, these were studies of the treatment of tardive dyskinesia in patients with stable schizophrenia, rather than of their effect on schizophrenia *per se*. Changes in diagnostic criteria over the last 60 or so years also hamper the drawing of clear conclusions.

Our synthesis found low rates of study heterogeneity. This is likely to be because of the small numbers of studies with similar effects sizes included in the meta-analysis. This result is a reflection, in some case, of the poor quality and reporting of the studies especially as four of the five studies included in the meta-analysis synthesis were from over 60 years ago. Grading of recommendations assessment, development and evaluation (GRADE) of the quality of evidence and strength of recommendation for the clinical outcome of tetrabenazine in schizophrenia and psychosis were informally assessed. For the assessment of quality of evidence for placebo-controlled studies slight or moderate improvement outcomes, two of the studies included in the meta-analysis were controlled trials although the randomisation was generally of poor quality and two studies were not randomised, risk of bias had some or high concerns, studies were relatively consistent versus placebo, participants were from a large range of ages and from a mostly inpatient population (apart from the study of Remington et al. (2012) which was conducted in outpatients), confidence intervals were wide or very wide for the placebo analyses indicating some imprecision, and formal analysis of publication bias was not possible given the small number of studies. Lastly, the inclusion of the study by Remington et al. may well diminish our effect size estimate as participants were largely resistant to standard treatments, including clozapine, and so were perhaps less likely to respond to VMAT-2 inhibitors.

The quality of evidence for this intervention, after examining the body of evidence, is low. The strength of recommendation is weak because the difference between desirable and undesirable effects is difficult to determine given the lack of formal assessment of adverse effects for most studies; the quality of evidence is low; and, at current rates, medication costs are higher overall for tetrabenazine than for standard treatment with second generation antipsychotics (National Institute for Health and Care Excellence 2023).

Adverse effects reported for tetrabenazine in included clinical trials as ‘very common’ were sedation, somnolence, parkinsonism and depression, whilst ‘common’ side effects were akathisia, anxiety and insomnia (Niemann and Jankovic 2019). Other adverse effects reported included dysphagia, hyperprolactinaemia and psychotic exacerbation (Solmi et al. 2018). Deutetrabenazine is probably better tolerated than tetrabenazine; adverse effects of deutetrabenazine, except for insomnia, are seen at a similar frequency to placebo (Niemann and Jankovic 2019; Solmi et al. 2018). Valbenazine appears well tolerated with patient completion rates in clinical trials similar to placebo (Solmi et al. 2018). It can commonly cause somnolence, headache, fatigue, dry mouth, vomiting and akathisia (Niemann and Jankovic 2019). Psychiatric side effects such as depression and suicidality have not been observed as being more common than with placebo in short- and longer-term clinical trials of patients with a stable mental state (Solmi et al. 2018). All VMAT-2 inhibitors are associated with QTc prolongation (Niemann and Jankovic 2019; Solmi et al. 2018). Tetrabenazine, at a dose of 50mg, prolongs QTc by an average of 8 milliseconds (AOP Orphan Ltd. 2020), deutetrabenazine (at 24mg) by 4.5 milliseconds (Teva Pharmaceuticals USA 2017) and valbenazine (at 80mg) by 2.1 milliseconds (Thai-Cuarto et al. 2018).

There are several limitations to note. The age of many of the studies examined means that we cannot be certain of outcomes (scales used were outdated, varied and numerous), diagnosis (little detail given; diagnostic criteria have long since changed) or even the morality of the studies (few details on patient consent provided). Even in our meta-analysis, we were forced to invent new somewhat subjective categories of response to account for the varied assessments used, and this inevitably affected our ability to interpret outcomes. In many studies, it is likely that participants were treatment-resistant, and this too affected outcome interpretation. Lastly, the vast majority of studies of psychosis used tetrabenazine, and findings from those studies may not generalise to deutetrabenazine or valbenazine.

Our analysis might have been improved by the inclusion of studies of reserpine (another VMAT inhibitor), or at least the number of studies available for inclusion might have increased. However, we had concluded *a priori* that reserpine lacked the close similarity in mode of action shared by the three VMAT inhibitors ultimately included. Reserpine is an irreversible inhibitor of VMAT-1 and VMAT-2 both centrally and peripherally and so is likely to have a different efficacy and tolerability profile.

None of these drugs is currently licensed for psychosis. Tetrabenazine is licensed in the EU for the treatment of ‘movement disorders due to Huntington’s chorea, hemiballismus, senile chorea, and related neurological conditions’ (AOP Orphan Ltd. 2020). It is not licensed in the EU for the treatment of schizophrenia even though it was initially developed as an antipsychotic agent (Schreiber et al. 1999) and, as this review shows, improves symptoms of psychosis. In the USA, deutetrabenazine is approved for chorea associated with Huntington’s disease and valbenazine for the treatment of TD. It is perhaps relevant to note that VMAT-2 inhibitors are not drugs of choice for psychosis in Huntington’s disease (Anderson et al. 2018).

The question that remains concerns the potential for VMAT-2 inhibitors to treat psychosis with a lower the risk of tardive dyskinesia and without the risk of inducing post-synaptic receptor supersensitivity. VMAT-2 inhibitors appear to be effective antipsychotics and, given that they are used to treat TD, we might assume that they are unlikely to cause this condition (although DA antagonists also ‘treat’ (mask) TD if the dose of DA antagonist is increased (Glazer and Hafez 1990)). Likewise, we might assume that, having little or no interaction with post-synaptic receptors, VMAT-2 inhibitors are unlikely to sensitise or upregulate these receptors. However, denervation of pre-synaptic dopamine neurones does induce postsynaptic supersensitivity (Kostrzewa and Brus 2016; Mandel et al. 1993). In addition to this, some VMAT-2 inhibitors do in fact have weak antagonist activity at post-synaptic dopamine receptors. Animal studies show that tetrabenazine can displace the D2 ligand at ^3^H-spiperone (Login et al. 1982), and severe dystonic reactions to high dose tetrabenazine are thought to be caused by dopamine receptor antagonism (Burke et al. 1985). Deutetrabenazine and valbenazine have little or no affinity for D2 receptors (Stahl 2018a), although a metabolite of deutetrabenazine ([-]-α-deuterated dihydrotetrabenazine) has moderate activity at D2 and D3 receptors (Brar et al. 2023). This antagonist activity at dopamine receptors may explain the very occasional cases of tardive dyskinesia reported in long-term tetrabenazine use (we could find only two in the literature) (LeWitt 2013; Palermo et al. 2020). The overall risk of TD is probably minimal: a long-term study of 448 people taking tetrabenazine for a range of movement disorders reported no cases of TD (Kenney et al. 2007). Reserpine, too, is only very rarely causatively linked to TD (Uhrbrand and Faurbye 1960). Neither valbenazine nor deutetrabenazine have been associated with emergent TD.

We do not know the optimal dose of any VMAT-2 inhibitor in treating psychosis, and we can therefore not be clear about the adverse effect burden of these drugs at that so far ill-defined dose. The dose-related nature of adverse effects offers the possibility of discovering doses that are both well tolerated and effective in psychosis.

We conclude that VMAT-2 inhibition deserves further scrutiny as a potential method for achieving antipsychotic efficacy with a potential for reduced risk of TD or dopamine receptor supersensitivity. Ultimately, however, there are insufficient data to unequivocally support or refute the efficacy and safety of VMAT-2 inhibitors to treat psychosis compared with placebo or active comparators, especially newer antipsychotics. Rigorous controlled trials which meet modern clinical trials standards are needed to answer this question definitively. Whether or not such trials are conducted may depend on the clinical utility of emerging non-DA antagonist antipsychotics (Tsapakis et al. 2023) and risk of TD associated with their long-term use.

### Supplementary information

ESM 1
