# Supplementary material for: Meta-analysis and systematic review of vesicular monoamine transporter (VMAT-2) inhibitors in schizophrenia and psychosis
Source: Psychopharmacology (Berl). 2024 Jan 19;241(2):225–41. doi: 10.1007/s00213-023-06488-3 (PMC10805984; doi:10.1007/s00213-023-06488-3)
Supplement: Supplementary file 1 — ESM 1 [file 213_2023_6488_MOESM1_ESM.docx]

*Supplementary Material*

*Meta-analysis and systematic review of vesicular monoamine transporter inhibitors in schizophrenia and psychosis*

Anne Conolly, Phoebe Wallman, Olubanke Dzahini, Oliver Howes and David Taylor

*Table of Contents*

| PRISMA checklist | 36 |
| --- | --- |
| PICOS | 39 |
| Specific search strings | 40 |
| Classification of improvement categorised to CGI-I | 44 |
| Details Risk of Bias concerns | 45 |
| Sensitivity analyses | 46 |
| Narrative review tables | 47 |

PRISMA checklist- Table S1

| **Section and Topic** | **Item #** | **Checklist item** | **Location where item is reported** |
| --- | --- | --- | --- |
| **TITLE** | | |  |
| Title | 1 | Identify the report as a systematic review. | 1 |
| **ABSTRACT** | | |  |
| Abstract | 2 | See the PRISMA 2020 for Abstracts checklist. | 2 |
| **INTRODUCTION** | | |  |
| Rationale | 3 | Describe the rationale for the review in the context of existing knowledge. | 3 |
| Objectives | 4 | Provide an explicit statement of the objective(s) or question(s) the review addresses. | 3 |
| **METHODS** | | |  |
| Eligibility criteria | 5 | Specify the inclusion and exclusion criteria for the review and how studies were grouped for the syntheses. | 4 |
| Information sources | 6 | Specify all databases, registers, websites, organisations, reference lists and other sources searched or consulted to identify studies. Specify the date when each source was last searched or consulted. | 4,5 |
| Search strategy | 7 | Present the full search strategies for all databases, registers and websites, including any filters and limits used. | 5,6, 36-39 |
| Selection process | 8 | Specify the methods used to decide whether a study met the inclusion criteria of the review, including how many reviewers screened each record and each report retrieved, whether they worked independently, and if applicable, details of automation tools used in the process. | 5-7 |
| Data collection process | 9 | Specify the methods used to collect data from reports, including how many reviewers collected data from each report, whether they worked independently, any processes for obtaining or confirming data from study investigators, and if applicable, details of automation tools used in the process. | 5 |
| Data items | 10a | List and define all outcomes for which data were sought. Specify whether all results that were compatible with each outcome domain in each study were sought (e.g. for all measures, time points, analyses), and if not, the methods used to decide which results to collect. | 6 |
|  | 10b | List and define all other variables for which data were sought (e.g. participant and intervention characteristics, funding sources). Describe any assumptions made about any missing or unclear information. | 6 |
| Study risk of bias assessment | 11 | Specify the methods used to assess risk of bias in the included studies, including details of the tool(s) used, how many reviewers assessed each study and whether they worked independently, and if applicable, details of automation tools used in the process. | 6 |
| Effect measures | 12 | Specify for each outcome the effect measure(s) (e.g. risk ratio, mean difference) used in the synthesis or presentation of results. | 6 |
| Synthesis methods | 13a | Describe the processes used to decide which studies were eligible for each synthesis (e.g. tabulating the study intervention characteristics and comparing against the planned groups for each synthesis (item #5)). | 6 |
|  | 13b | Describe any methods required to prepare the data for presentation or synthesis, such as handling of missing summary statistics, or data conversions. | 6 |
|  | 13c | Describe any methods used to tabulate or visually display results of individual studies and syntheses. | 6,7 |
|  | 13d | Describe any methods used to synthesize results and provide a rationale for the choice(s). If meta-analysis was performed, describe the model(s), method(s) to identify the presence and extent of statistical heterogeneity, and software package(s) used. | 6 |
|  | 13e | Describe any methods used to explore possible causes of heterogeneity among study results (e.g. subgroup analysis, meta-regression). | NA |
|  | 13f | Describe any sensitivity analyses conducted to assess robustness of the synthesized results. | 7 |
| Reporting bias assessment | 14 | Describe any methods used to assess risk of bias due to missing results in a synthesis (arising from reporting biases). | 6 |
| Certainty assessment | 15 | Describe any methods used to assess certainty (or confidence) in the body of evidence for an outcome. | 6 |
| **RESULTS** | | |  |
| Study selection | 16a | Describe the results of the search and selection process, from the number of records identified in the search to the number of studies included in the review, ideally using a flow diagram. | 7 |
|  | 16b | Cite studies that might appear to meet the inclusion criteria, but which were excluded, and explain why they were excluded. | 8, 18 |
| Study characteristics | 17 | Cite each included study and present its characteristics. | 9-11 |
| Risk of bias in studies | 18 | Present assessments of risk of bias for each included study. | Table 2 & S3 page 14, 41 |
| Results of individual studies | 19 | For all outcomes, present, for each study: (a) summary statistics for each group (where appropriate) and (b) an effect estimate and its precision (e.g. confidence/credible interval), ideally using structured tables or plots. | 13-17 |
| Table 2,3 and S3, page 17, 45  Results of syntheses | 20a | For each synthesis, briefly summarise the characteristics and risk of bias among contributing studies. | Table 2,3 & S3 page 14, 41 |
|  | 20b | Present results of all statistical syntheses conducted. If meta-analysis was done, present for each the summary estimate and its precision (e.g. confidence/credible interval) and measures of statistical heterogeneity. If comparing groups, describe the direction of the effect. | 12 |
|  | 20c | Present results of all investigations of possible causes of heterogeneity among study results. | NA |
|  | 20d | Present results of all sensitivity analyses conducted to assess the robustness of the synthesized results. | 12, 42 |
| Reporting biases | 21 | Present assessments of risk of bias due to missing results (arising from reporting biases) for each synthesis assessed. | 12,42 |
| Certainty of evidence | 22 | Present assessments of certainty (or confidence) in the body of evidence for each outcome assessed. | 6-7,25 |
| **DISCUSSION** | | |  |
| Discussion | 23a | Provide a general interpretation of the results in the context of other evidence. | 25-27 |
|  | 23b | Discuss any limitations of the evidence included in the review. | 25-27 |
|  | 23c | Discuss any limitations of the review processes used. | 25-27 |
|  | 23d | Discuss implications of the results for practice, policy, and future research. | 27 |
| **OTHER INFORMATION** | | |  |
| Registration and protocol | 24a | Provide registration information for the review, including register name and registration number, or state that the review was not registered. | Not registered |
|  | 24b | Indicate where the review protocol can be accessed, or state that a protocol was not prepared. | Not prepared |
|  | 24c | Describe and explain any amendments to information provided at registration or in the protocol. | NA |
| Support | 25 | Describe sources of financial or non-financial support for the review, and the role of the funders or sponsors in the review. | NA |
| Competing interests | 26 | Declare any competing interests of review authors. | As declaration of interest page 1 |
| Availability of data, code and other materials | 27 | Report which of the following are publicly available and where they can be found: template data collection forms; data extracted from included studies; data used for all analyses; analytic code; any other materials used in the review. | Supplementary material and authors |

*PICOS*

Population

- Adults aged 18 and over, including older adults aged more than 65

Problem

- Psychotic disease or schizophrenia
- Definition of these conditions in older studies may differ so this section may require amendment as these terms may not have been used at that time

Intervention

- Treatment of psychosis or schizophrenia in patients with dopamine synthesis inhibitors (i.e. tetrabenazine, deutetrabenazine, valbenazine)

Comparison

- Placebo or/& antipsychotic drug

Outcome

- Efficacy
  - The Brief Psychiatric Rating Scale (BPRS) change
  - Clinician assessment
- Adverse effects
  - Extrapyramidal side effects (EPSE) by rating scale or clinician assessment

Studies

- Randomised controlled trails
- Non-randomised studies

*Searches*

Embase, 09/11/2020

Database: Embase <1974 to 2020 Week 45>

Search Strategy:

--------------------------------------------------------------------------------

1 Schizophrenia.mp. or exp schizophrenia/ or exp schizophrenia spectrum disorder/ (211092)

2 Psychosis.mp. or exp psychosis/ (298029)

3 1 or 2 (315102)

4 Valbenazine.mp. or exp valbenazine/ (247)

5 Deutetrabenazine.mp. or exp deutetrabenazine/ (276)

6 Tetrabenazine.mp. or exp tetrabenazine/ (3350)

7 "Dopamine synthesis inhibitor*".mp. (38)

8 "Vesicular monoamine transporter".mp. or exp vesicular monoamine transporter/ (4304)

9 "Vesicular monoamine transporter inhibitor*".mp. (25)

10 4 or 5 or 6 or 7 or 8 or 9 (7561)

11 3 and 10 (671)

12 limit 11 to human (586)

***************************

**01/04/21**

“1-9569”

Embase Classic, 22/10/2020

1. schizophrenia/ or exp psychosis/ or exp schizophrenia spectrum disorder

2. schizophrenia.mp.

3. psychosis.mp.

4. 1 or 2 or 3

5. valbenazine/ or exp dopamine receptor affecting agent/

6. valbenazine.mp. or exp valbenazine/

7. deutetrabenazine/ or exp dopamine receptor affecting agent/

8. deutetrabenazine.mp. or exp deutetrabenazine/

9. dopamine synthesis inhibitor*.mp.

10. dopamine transporter inhibitor*.mp.

11. tetrabenazine.mp. or exp tetrabenazine/

12. 5 or 6 or 7 or 8 or 9 or 10 or 11

13. 4 and 12

14. limit 13 to human

01/04/21

“1-9569”

Medline, 22/10/2020

1. exp "schizophrenia spectrum and other psychotic disorders"/ or psychotic disorders/ or exp schizophrenia/

2. exp Psychotic Disorders/

3. 1 or 2

4. tetrabenazine.mp. or exp Tetrabenazine/

5. valbenazine.mp.

6. deutetrabenazine.mp.

7. dopamine synthesis inhibitor*.mp.

8. vesicular monoamine transport*.mp.

9. dopamine transporter inhibitor*.mp.

10. 4 or 5 or 6 or 7 or 8 or 9

11. 3 and 10

12. limit 11 to humans

01/04/21

“1-9569”

PsycInfo, 28/10/2020

Database: APA PsycInfo <1806 to October Week 3 2020>

Search Strategy:

--------------------------------------------------------------------------------

1 exp Schizophrenia/ or Schizophrenia.mp. (134726)

2 exp Psychosis/ or Psychosis.mp. (133923)

3 1 or 2 (168946)

4 Valbenazine.mp. (29)

5 Deutetrabenazine.mp. (30)

6 exp Tetrabenazine/ or Tetrabenazine.mp. (398)

7 "Dopamine synthesis inhibitor*".mp. (4)

8 "Vesicular monoamine transporter".mp. (375)

9 "Vesicular monoamine transporter inhibitor*".mp. (5)

10 4 or 5 or 6 or 7 or 8 or 9 (721)

11 3 and 10 (64)

12 limit 11 to human (57)

***************************

01/04/21

“1-9569”

Psychiatry Online, 29/10/2020

(schizo* OR "dementia praecox" OR psycho?s OR psychotic) AND (valbenazine OR deutetrabenazine OR tetrabenazine OR "dopamine synthesis inhibitor*" OR "vesicular monoamine transporter" OR "vesicular monoamine transporter inhibitor*")

01/04/21

“1-9569”

Pubmed, 01/04/2021

(schizo* OR "dementia praecox" OR psychos?s OR psychotic) AND (valbenazine OR deutetrabenazine OR tetrabenazine OR "dopamine synthesis inhibitor*" OR "vesicular monoamine transporter" OR "vesicular monoamine transporter inhibitor*" OR nitoman OR 1-9569)

Web of Science, 28/10/20


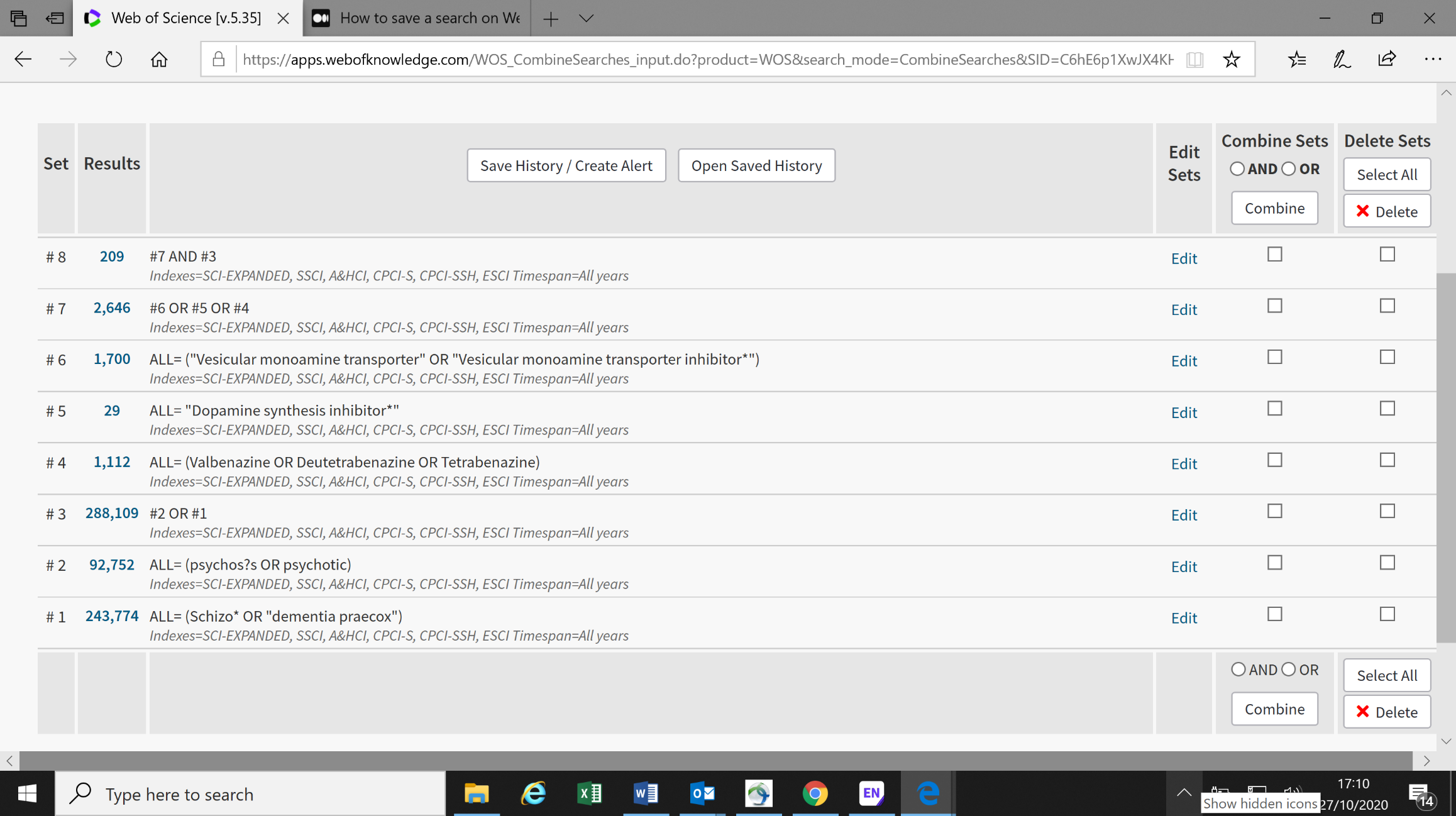


01/04/21

“1-9569”

Top 10% of Web of Science, 28/10/20

1. Howes et al., 2012- 110 references (108 downloaded)
2. Ford, 2014- 143 references (142 downloaded)
3. Cousins et al., 2009- 258 references (251 downloaded)
4. Howes et al., 2017- 134 references (133 downloaded)
5. Tabares-Seisdedos et al., 2009- 420 references (415 downloaded)
6. Adell et al., 2004- 287 references (279 downloaded)
7. Gubernator et al., 2009- 23 references
8. Morales et al., 2014- 76 references (73 downloaded)
9. Rigdon et al., 1992- 50 references (49 downloaded)
10. Sulzer et al., 2016- 462 references (458 downloaded)
11. Mestre et al., 2009- 113 references (105 downloaded)
12. Weinstein et al., 2017- 129 references (128 downloaded)
13. Kumakura et al., 2007- 57 references
14. Bhidayasiri et al., 2013- 40 references (36 downloaded)
15. Bonelli et al., 2006- 291 references (283 downloaded)
16. Halpin et al., 2014- 168 references (167 downloaded)
17. Wimalasena et al., 2011- 166 references (161 downloaded)
18. Ashok et al., 2017- 107 references (105 downloaded)
19. Hauser et al., 2017- 25 references (23 downloaded)
20. Stahle, 1992- 111 references (95 downloaded)
21. Mooslehner et al., 2001- 37 references (33 downloaded)

*Table S2 Classification of improvement categorised to CGI-I*

| Study ID | Outcome measure | Response classification |
| --- | --- | --- |
| Ashcroft 1961(Ashcroft et al. 1961) | Baker and Thorpe, clinical assessment | Definite improvement', 'significant improvement', 'very marked improvement in thought disorder', example case study described = CGI-I 2 |
| Lingjærde 1963(Lingjaerde 1963) | Clinician assessment | 'Marked improvement' = CGI-I 2; 'slight improvement' = CGI-I 3, case study described by authors  Unable to extract data for moderate improvers for the first period’ only slight improvers included in analysis |
| Remington 2012(Remington et al. 2012) | Brief Psychiatric Rating Scale (BPRS), Clinical Global Impression (CGI), Global Assessment of Functioning (GAF), Behaviorally Anchored Rating Scale (BARS), Riker Sedation-Agitation Scale (SAS), Calgary Depression Scale for Schizophrenia (CDS) | CGI measured directly in this study  20% improvement in total BPRS scores from baseline to end point. We classified this as CGI 3 = minimally improved (Leucht et al. 2005) |
| Smith 1960(Smith 1960) | Clinician assessment, Behavioural rating scale | 'Excellent response', 'moderate response' = CGI-I 2; 'slight response' = CGI-I 3 |
| Weckowicz 1960(Weckowicz et al. 1960) | Weyburn assessment scale, clinician assessment | 'Slight improvement' = ~~CGI-I 2~~ CGI-I 3 |

*Table S3 Details of Risk of Bias Concerns*

| *Study* | *Domain* | *Elaboration* | *Outcome* |
| --- | --- | --- | --- |
| Ashcroft et al.1961(Ashcroft et al. 1961) | Randomisation process | The allocation sequence was not random; groups were matched by a colleague. No information on whether the allocation sequence was concealed until participants were enrolled and assigned to interventions. | Some concerns |
|  | Selection of the reported result | No pre-specified analysis plan. | Some concerns |
| Lingjaerde 1963(Lingjaerde 1963) | Deviations from intended interventions | The code was broken in cases where complications necessitated an interruption of treatment. No information on whether important non protocol interventions were balanced across intervention groups. No information on whether an appropriate analysis was used to estimate the effect of adhering to the intervention. | High risk |
|  | Selection of the reported result | No pre-specified analysis plan. Assessed at baseline and then weekly but only final result given. Scale was vague: worsening, no definite change, slight improvement, marked improvement, complete remission. | Some concerns |
| Smith 1960(Smith 1960) | Randomisation process | The allocation sequence was not random; groups were matched. No information on whether the allocation sequence was concealed until participants were enrolled and assigned to interventions. No information on baseline differences. | Some concerns |
|  | Deviations from intended interventions | No information on whether participants, carers or people delivering the intervention were aware of the participants’ assigned intervention during the trial. No information on whether important non protocol interventions were balanced across intervention groups. No information on whether an appropriate analysis was used to estimate the effect of adhering to the intervention. | High risk |
|  | Measurement of the outcome | No information on whether the outcome assessors were aware if the intervention received by study participants and the assessment of the outcome could have possibly been influenced by knowledge of the intervention received. | Some concerns |
|  | Selection of the reported result | No pre-specified analysis plan. Did not state when the “clinical response” was assessed. | Some concerns |
| Weckowicz et al. 1960(Weckowicz et al. 1960) | Randomisation process | The allocation sequence was not random; groups were matched. No information on whether the allocation sequence was concealed until participants were enrolled and assigned to interventions. No information on baseline differences. | Some risk |
|  | Measurement of the outcome | No information on whether the outcome assessors were aware if the intervention received by study participants and the assessment of the outcome could have possibly been influenced by knowledge of the intervention received. | Some concerns |
|  | Selection of the reported result | No pre-specified analysis plan. | Some concerns |

*Sensitivity analyses*


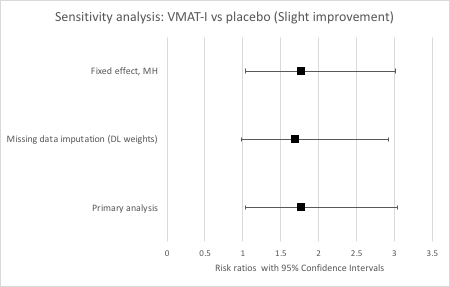


*Fig* *S1*

*
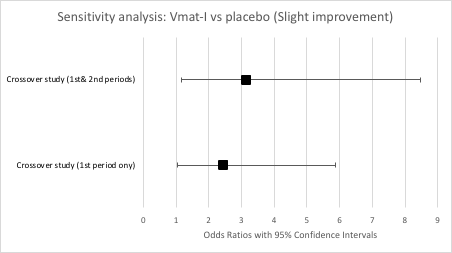
*

*Fig S2*

*Narrative synthesis extended*

*Table S4 Tetrabenazine studies:*

| *Author(s)/year:* | *Previous &/or concurrent treatment (not including ADR treatments for TBZ/ VBZ treatment)* | *Comparator (dose)* | *Comparator outcome* | *Adverse effects* | *Comments* |
| --- | --- | --- | --- | --- | --- |
| Voelkel (1958)(Voelkel 1958) | 'Patients with repeated acute attacks after previous good remissions or with acute initial illnesses', patients *may* have been having concurrent ECT - unclear, case series 2 used MAOI antidepressant iproniazid pretreatment then TBZ despite opposing actions | NA | "[Iproniazid alone] loosening of sad moods, increasing interest, a decrease in contact inhibition and relieving feelings of guilt, insufficiency, ideas and self-reproaches." | More than 450mg/day for 4-6 days = akinetic-abulic syndrome with fine finger tremors, extremity rigors, stiffness in facial expressions and gestures, hypokinesis and bradykinesia but not at doses up to max of 180mg daily; slight increase in body weight, extracellular fluid volume decreases slightly, no jaundice or allergy, heart or circulatory conditions or resting blood pressure, less sedative than reserpine, does not prolong postconvulsive apnoea when given with ECT | Translation from German |
| Haug & Stenstad (1959)(Haug 1959) | Not stated in abstract in Lingjaerde 1963 | NA | NA | 1 patient = stupor-like condition with EPSE 1 (reinstated 2 weeks after withdrawal of TBZ); 'insignificant fall in BP in a few patients', EPSE; sedative | Data from abstract in Lingjaerde 1963, could not get full text from British Library |
| Lingjaerde (1959)(Lingjaerde 1959) | Yes group 1 (pre-treatment with reserpine) and group 2 ('7 with reserpine, of which only 2 with possible effect; 6 with Trilafon (perphenazine) of which 1 with effect; 2 with chlorpromazine, of which 1 with effect). This group therefore consists for a large part of patients who have previously reacted poorly to ataractica (i.e. tranquiliser) treatment, and 'to that extent constitute a negative sample'). | Group 2 treated with TBZ as group 1 but then stopped and reserpine started (dose of reserpine not stated) | Not stated | Anorexia possibly nausea = 5, slackness = 2, restlessness = 4 (after dose increase, resolved on dose reduction, some needed <100mg dose), increased appetite = 1 (subjective side effects). 4/5 patients with anorexia pre-treatment with reserpine and ADR occurred at start of TBZ treatment so could be reserpine withdrawal, this ADR spontaneously resolved without dose change of TBZ. Group 1 (pre-treatment with reserpine) raised BP esp diastolic and pulse, group 2 less change in BP and pulse - possible lower risk of bradycardia with TBZ than reserpine?. Transaminases and leucocytes normal (for 9 patients); no EPSE (but restlessness reported). Authors report fewer side effects for TBZ than phenothiazines (but not measured in this study). | Translation from Norwegian. No available response data for reserpine treatment period. Also 20 days data after stopping tetrabenazine does not count as placebo treatment. |
| Voelkel & Dresler (1959)(Voelkel and Dressler 1959) | ECT concurrent treatment in cases 5 and 6 with catatonia; case no 1 previous treatment with insulin shock therapy, phenothiazines, ECT, iminodibenzyl; case 2 concurrent insulin course; case 3 previous unsuccessful treatment with iminodibenzyl, case 4 previous phenothiazine therapy achieving only moderate psychomotor calming and improved contact; case 6 pre-treatment with psychotropic substances (phenothiazines, reserpine) was just as unsuccessful as insulin shock therapy, electrical and Cardiazole treatments; case 7 phenothiazine and electroconvulsive treatment failed; case 8 previous unsuccessful treatment with phenothiazines inc iminodibenzyl, iproniazid, serine isopropylhydrazide, case 11 convulsive and neuroleptic therapy bring only short-term lightening, a reserpine treatment lasting several months also reduces the symptoms, which are worsened in terms of content, only for a shorter time. | NA | NA | Hb, ESR, urinary bile, FBC, normal; no jaundice; BP (inc. orthostatic) and pulse normal; EEG normal; no photosensitivity, micturition disorders, constipation, diarrhoea; no hypersecretion irritation or inflammation of GIT; dry mouth, fine tremor at higher doses (> 150mg); EPSE at 450mg; ECT - no effect on threshold, intensity, duration of phases. | Translation from German |
| Flegel (1960)(Flegel 1960) | 'For the most part have been treated in vain for years or with only temporary or poor success' 'Nine of the well-improved cases were treated with phenothiazines and reserpine three in vain, some immediately before and some during the previous year. Six of the partial remissions could have been favoured by a partial success of the pre-treatment.' 'Other cases that responded to Nitoman had also reacted favourably to reserpine and phenothiazines.' | NA | NA | Rigidity 16; tiredness 11; tremor 6; BP fall 6; weight loss 6; slight reduction in haemoglobin 4; confusion 2; fever 3; pneumonia 1 (possibly secondary to parkinsonism) | Translation from German |
| Heinze (1960)(Heinze 1960b) | 'Whenever possible, no combination treatment was carried out in order to determine the selective effect of the drug.' 'Only in individual cases was an appropriate additional medication administered at the same time to prevent Parkinson's syndromes.' 'In catatonic, hebephrenic and paranoid-hallucinatory forms of schizophrenia' 'cases we were forced to switch to preparations from the perazine group or to carry out an electric shock treatment.' 'a combination of the preparation with simultaneous therapeutic spasms was refrained from whenever possible.' ’High percentage of refractory patients’- from Lingjaerde | NA | NA | Inner restlessness 3, Parkinsonian-like effect 5, opisthotonos 1; no effect on heart or circulatory system, BP, LFTs | Translation from German |
| Lende (1960)(Lende 1960) | 'Unresponsive to previous treatment with tranquillisers', 'many refractory to other medication', 1 patient previous/concurrently treated with thioridazine (unclear if stopped when started TBZ); 1 patient previously treated with CPZ | NA | NA | On high dose lethargy, trembling, muscle rigidity, drooling; one patient exhibited parkinsonian symptoms (effects considered to be signs of overdosage); no cases of blood or liver damage | None |
| Montagut (1960)(Montagut 1960) | Not stated | NA | NA | Sedation 3; restlessness 3; diarrhoea 2; vomiting 1. Six patients with neuroses very troubled by side effects | Data from abstract in Lingjaerde 1963, conference proceedings |
| Narros & Carranza (1960)(Narros Martin 1960) | Not stated | NA | NA | Somnolence; general discomfort; coated tongue; anorexia; loss of weight; parkinsonism (tremor; sialorrhea, back pain. No changes in blood tests, liver function, pulse, temperature or BP | Data from abstract in Lingjaerde 1963, conference proceedings |
| Pomme et al (1960)(Pomme et al. 1960) | Unclear if concurrent or pre-treatment- patients with schizophrenia = “sleep cure” (intramuscular injection Laborit's twice daily, and oral chlorpromazine) no case numbers stated; hallucinatory syndromes= case 5 sleep cure before TBZ 'stabilising effect', seismotherapy 2 cases (three electric shocks, one each day, three days in a row, for example), CPZ 4 cases. | NA | NA | EPSE, EPSE with dystonia in patients with hallucination syndrome and schizophrenia; GI = mild dyspepsia and anorexia, constipation, 1 case of vomiting preventing continued treatment; CV = BP, some hypotension, some hypertension, paroxysmal tachycardia 4 cases, extrasystole 1 case; skin = no ADRs | Translation from French |
| Reda & Germano (1960)(Reda and Germano 1960) | Out of 28 cases treated with tetrabenazine, 13 had never had any ‘neuroplegic’ drug treatment; of these 13 cases, all delusional syndromes, 7 were acute forms and 6 chronic. The remaining 15 cases (10 delusional syndromes, 5 psychoneuroses) had already been previously treated with ‘neuroplegics’, such as reserpine and chlorpromazine for periods ranging from 2 months to 4 years with results ranging from poor to fairly good.  With reserpine and chlorpromazine the case numbers 4, 5, 9, 12, 28 had obtained fairly positive results; the case numbers 7, 10, 18, 19, 22, 24, poorly positive and no. 27 negative result. One case, the case number 15, who presented with a paraphrenic delusional syndrome, arising after meningococcal meningitis, after the failure with tetrabenazine and was treated with other neuroplegic drugs, even with negative results and improved somewhat only after an ECT course; the same situation occurred with case number 17, 'an obsessive psychoneurosis improved only after ECT' | NA | NA | Somnolence 'in some cases'; tremor 2; paroxysmal trismus 1; vertigo ; motor activity 1; inability to remain still 1; insomnia 1; confusion 1; no changes in blood tests (FBC, glycaemia, LFTs), BP, pulse, menstrual cycle, urination, bowel function, breathing | Translation from Italian |
| Sacerdoti (1960)(Sacerdoti 1960) | One schizophrenic patient pre-treated with Marsilid (iproniazid) for whom reserpine or iproniazid alone were ineffective | NA | NA | Drowsiness 7/26, mild lowering of arterial pressure 2/26 (1 initially elevated), restlessness 1/26, tremors 2/26, psychomotor slowdown 1/26, bradycardia 1/26, transitory astenia 1/26, vertigo 1/26, nausea 1/26, drooling 1/26, retching 1/26. Unchanged EEG, no appreciable changes in cardiovascular, digestive or urinary systems. | Translation from Italian |
| Schmitt (1960)(Schmitt 1960) | Not stated | NA | NA | Slight EPSE | Translation from German |
| Smith & Stockhausen (1960)(Stockhausen 1960c) | Not stated | NA | NA | Hypotension in high doses, akinetic-abulic syndrome no numbers stated, lactation in some women; no effect on FBC, LFT, sedimentation reaction, GI disturbance, respiration and heart rate. | Translation from German |
| Stockhausen (1960b)(Stockhausen 1960a) | 1 case previously treated with ECT and phenothiazines with some effect; 1 case previously treated with ECT and reserpine with no effect | NA | NA | Parkinson-like syndrome in around 85% of cases (when dose was higher than 50mg t.i.d.), about 10% profuse sweating, 1 case of paresthesia, over 20% metrorrhagias and 5 cases of spontaneous lactation, non-significant Hypotension, liver toxicity, allergy, GI disorder, dyscrasias | None |
| Stockhausen (1960c)(Stockhausen 1960b) | Not stated | NA | NA | No side effects on blood count, sedimentation reaction, LFT, GI upset and no allergies. 2 cases petechiae, hypotension in high doses, akinetic-abulic syndrome no numbers stated, EEG changes 50% (alpha activation 1/4 of patients, fatigue pattern 1/4 of patients), menstrual disturbance and lactation in some women. | Translation from German |
| Stumpf (1960)(Stumpf 1960) | Not stated | NA | NA | EPSE 7, case lockjaw 1, inner restlessness 4 | Translation from German, 'low success rate because treatment terminated prematurely' and severe cases |
| Bertolotti (1961)(Bertolotti and Munarini 1961) | 'Most of these had already been subjected to all or almost all of the shock treatments and the most recent psychopharmacological therapies, some even to prefrontal lobotomy, with no apparent results or with only transient results' | NA | NA | Transient asthenia and drowsiness, slight hypotension; slight change in LFTs 1 case; no EPSE even at high doses; mild sleepiness; no GI disturbances; drooling & yawning 1 case; NA ADRs, BP, FBC, LFT, EPSE | Translation from Italian |
| Borenstein et al (1961)(Borenstein et al. 1961) | 'Throughout the therapeutic trials, no other psychotropic substance was associated.' | NA | NA | Total adverse effects EPSE 38 (9 fine tremor, 10 akathisia, 1 difficulty swallowing, 3 painful contraction of the masticatory muscles); insomnia 39; hypersomnia in 1/3rd of cases at start of treatment; psychomotor excitation 4; asthenia 1; epileptiform seizure 1; itching (no rash) 2; inguinal eczema 2; nausea and vomiting 2; slight BP decrease (no numbers); blood tests done on 12 patients (blood sugar, urea, sedimentation rate) normal | Translation from French |
| Espinosa (1961)(Espinosa 1960) | Some previously treated with ECT (some concurrently) and reserpine | NA | NA | Parkinsonian symptoms 3 (at 150mg responded to dose decrease), diarrhoea 5 managed by 50% dose reduction or symptomatic treatment) , vomiting 1, orthostatic dizziness 2, heavy head or suffocation or sensations in body 'some patients during first days' , no drowsiness, changes in pulse or temperature or blood tests | Translation from Spanish |
| Lustig (1961)(Lustig 1961) | Combined with sleep treatment, insulin coma or ECT (18 patients) for patients with SCZ | NA | NA | Parkinsonism 17; acute extrapyramidal paroxysms 4. No changes in BP, no GI disturbances, no irritation at injection site | Data from abstract in Lingjaerde 1963, conference proceedings |
| Singer et al (1961)(Singer 1961) | Not stated | NA | NA | Fall in BP initially and on dose increase, tachycardia and palpitations 7, dryness of mouth 'initially in almost everybody', hypersalivation 4, frequent akathisia, somnolence, insomnia. Also observed parkinsonism, 'neuroleptic syndrome 1', anxiety, constipation. | Abstract from Lingjaerde 1963, from BL, may be duplicate or partial report of Kammerer 1962 |
| Kammerer et al (1962)(Kammerer et al. 1962) | '4 patients had previously been subjected to chlorpromazine' | NA | NA | Moderate hypotension with dizziness and malaise but no collapse; tachycardia & palpitations ; dryness of mouth in ‘almost everybody'; hypersalivation 'half of patients'; constipation & anorexia ' a few patients'; nausea and vomiting due to appendicitis; weight gain; drowsiness and fatigue especially at 150mg dose; nocturnal insomnia was frequent; allergic epidermal desquamation 1; akathisia; parkinsonism; 'neuroleptic syndrome' 4; oculogyric crisis 1 anxiety; blood tests = urea decrease, increase blood sugar, LFTs normal, neutrophils reduced & increase in lymphocytes, small decrease in platelets and fibrinogen; no ECG changes or renal impairment | Translation from French |
| Shimizu et al (1962)(Shimizu et al. 1962) | ECT concurrent treatment in 4 cases (3/6 of good improvement group) | NA | NA | Not stated | Translation from Japanese |
| Burckard et al (1962)(Burckard et al. 1962) | 1 case previous treated unsuccessfully with reserpine (Serpasil) | NA | NA | Women sleep disturbance 10; restless legs 4, tremor no case numbers listed. Men increased anxiety 4 (TBZ stopped in 2 cases and continued in 2), sleep disturbance (day time sleepiness & night time insomnia) 8, impatience and pain in lower limbs 5, EPSE 6; men and women - weight gain (average 2kg), BP, FBC, LFT normal. | Translation from French, authors note some clinical benefit from renewed staff attention |
| Cwynar et al (1962)(Cwynar et al. 1962) | Yes "previously with other commonly used agents"; 1 case 1st episode | NA | NA | At 300mg a day, 5 no side effects, 9 'felt very unpleasant', 3 stopped further treatment, anxiety and insomnia 'most people', seizures 1, Parkinsonoid syndrome with significant limb stiffness, drooling and masking facial expressions 1, motor restlessness 3, severe insomnia 2. athetotic movements and painful excitement movements 2 (at 150mg or lower 1 of whom ended with a grand mal-like attack). No CV, GI, haematological, urinary system ADRs. | Translation from Polish |
| Brauchitsch (unpublished, pre-1963)(Brauchitsch) | Not stated | Placebo substitution in 23 patients | 23 patients given PBO after TBZ, 14 relapsed | ‘Tetrabenazine-malaise’ i.e. dysphoria, tiredness, tension, nausea, fluttering vision, lethargy, irritability almost all patients lasted for 1 week. Bradycardia 61; hypothermia (55); parkinsonism 53; sleep disturbance 28; turbulence 19 (restlessness, compulsive walking, strong inner tension increasing to confusion); torsion spasm, opisthotonos, oculogyric crises 5; athetosis-like spasm 3; vasovagal syncope 5; depression 5; 2 patients died (cardiac insufficiency, malignant catatonia (pre TBZ treatment)). 25% of stopped treatment because of ADRs. No fall in BP, GI disturbance, constipation, allergic skin reactions, injection site pain, weight gain, hematopoietic or hepatic disturbances. | Article in German. Data here are from summary in Linjaerde 1963. Includes 13 patients <20yrs, this may be an exclusion if the patients in question are <18yrs. |
| Matsumoto et al (1966)(Matsumoto et al. 1966) | 'Electrical therapy, insulin impact therapy, chlorpromazine and other special drug therapy has been performed', promethazine used for EPSE; 1 case previous treatment with ECT, CPZ, levomepromazine, chlorpothixene in succession. | NA | NA | Anxiety, impatience (39%), Parkinsons's syndrome (22.4%), all 3 common side effects treated with promethazine. ADRs more likely if dose > 150mg. Palpitations 1 case, chest discomfort, orthostatic hypotension, Insomnia, hypertonia, torticollis, protruding tongue, wandering, restlessness | Translation from Japanese. |
| Kalian et al (1993)(Kalian et al. 1993) | Haloperidol, penfluridol, sulpiride. TBZ added to clozapine 200mg | NA | NA | Not stated | Tetrabenazine for tardive dyskinesia in patient on clozapine |
| Gordon et al (1998)(Gordon et al. 1998) | First generation antipsychotic up to 3 x maximum BNF doses, risperidone, ECT, clozapine | NA | NA | BP and ECG normal | Patient on depot antipsychotic, risperidone and diazepam 15mg (started at same time as TBZ, authors acknowledge that could have reduced agitation). Advised monitoring BP as can cause hypotension. |

GI= gastrointestinal, LFT= liver function tests, FBC= full blood count, CV= cardiovascular, EPSE= extrapyramidal side effects, BP= blood pressure, ECT= electroconvulsive therapy

*Table S5 Valbenazine studies:*

| Author(s)/year: | Previous &/or concurrent treatment (not including ADR treatments for TBZ/ VBZ treatment) | Comparator (dose) | Comparator outcome | Adverse effects | Comments |
| --- | --- | --- | --- | --- | --- |
| Hauser et al (2017)(Hauser et al. 2017) | 194/227 (85%) of patients were receiving concurrent antipsychotics (some patients were receiving more than 1 antipsychotic) | placebo | Placebo PANSS total change from baseline to week 6 = -1.5 points | Somnolence 5.3% (PBO 3.9%), akathisia 3.3% (PBO 1.3%), dry mouth 3.3% (PBO 1.3%), suicidal ideation 2.6% (PBO 5.3%) (also arthralgia, headache, vomiting, dyskinesia, anxiety, insomnia, fatigue, urinary tract infection, weight gain) | KINECT 3 (study 1304) data supplement table S2, participants had 'stable psychiatric status', excluded in BPRS >=50 or PANSS total >=70. Baseline BPRS ~ 30. |
| Josiassen et al (2017)(Josiassen et al. 2017) | stable doses of concomitant medications for psychiatric disorder were permitted throughout the studies' | Placebo for 2 of 3 pooled studies but this data not included in comparator analyses | NA | ADRs in 66.5% of patients. UTI 6.1%, headache 5.8%, somnolence 5.2%, suicidal ideation 4.5% (fatigue, dizziness, diarrhoea, constipation, anxiety, depression, vomiting, nausea, back pain, bronchitis and arthralgia) | Pooled analysis of 3 studies (2 of which contained a placebo arm KINECT (study 1201) & KINECT 3(study 1304)). KINECT 3 (study 1304) is Josiassen 2017. KINECT (study 1201) data is not available as mentioned in email message from Neuroscience company. |
| Lindenmayer (2017)(Lindenmayer et al. 2017) | >40% had concomitant medications- most common being antipsychotics, antidepressants and anxiolytics | Placebo | PANSS change after 6 weeks PBO -2.6 | Suicidal ideation/behaviour VBZ 5.9% vs. PBO 2% at baseline; weeks 2-8 VBZ 5.9% PBO 0% | From conference abstract, KINECT 2 study (study 1202) |
| Lindenmayer (2019)(Lindenmayer et al. 2019) | Stable concomitant psychiatric medications were allowed | NA | NA | Most common: Somnolence 7%, headache 7% in schizophrenia group | From conference abstract, KINECT 4 (study 1402) one of the studies in in the pooled analysis by Josiassen 2017 |
| Khurram et al (2021)(Khurram et al. 2021) | Clozapine concurrently.  Previously aripiprazole, risperidone, | NA | NA | Not reported | Case report |
| Lindenmayer et al (2022)(Lindenmayer et al. 2022) | Previously quetiapine, haloperidol, lurasidone, clozapine | NA | NA | Not reported | Case report |
